# Effect of Grape Marc Added Diet on Live Weight Gain, Blood Parameters, Nitrogen Excretion, and Behaviour of Sheep

**DOI:** 10.3390/ani12030225

**Published:** 2022-01-18

**Authors:** Huichu Wu, Pangzhen Zhang, Fan Zhang, Md Safiqur Rahaman Shishir, Surinder S. Chauhan, Innocent Rugoho, Hafiz Suleria, Guangyong Zhao, Brendan Cullen, Long Cheng

**Affiliations:** 1 Faculty of Veterinary and Agricultural Sciences, The University of Melbourne, Victoria 3647, Australia; huichuwu0023@gmail.com (H.W.); pangzhen.zhang@unimelb.edu.au (P.Z.); zf_rachel@163.com (F.Z.); shishir.an@bau.edu.bd (M.S.R.S.); ss.chauhan@unimelb.edu.au (S.S.C.); hafiz.suleria@unimelb.edu.au (H.S.); bcullen@unimelb.edu.au (B.C.); 2 Department of Animal Nutrition, Bangladesh Agricultural University, Mymensingh 2202, Bangladesh; 3 Lely Australia Pty Ltd., 84 Agar Drive, Truganina, VIC 3029, Australia; irugoho@lely.com; 4 College of Animal Science and Technology, China Agricultural University, Beijing 100083, China; zhaogy@cau.edu.cn

**Keywords:** grape pomace, ruminant, processing by-products, nitrogen use efficiency, phytochemicals

## Abstract

**Simple Summary:**

This experiment explored how feeding grape wine production waste product grape marc impacts on sheep production. Forty merino sheep were divided into two groups; one group received a sheep industry standard diet (control), and one group received a treatment diet which had 20% of the control diet replaced by grape marc. The results showed that the grape marc diet led to a higher intake and faecal nitrogen/urinary nitrogen ratio, but no difference in sheep live weight gain, behaviour, and parasitic egg count compared with control diet-fed sheep. Overall, the results showed that feeding grape wine production waste product grape marc as a cheap feed, to replace 20% of the control ration, can maintain sheep productivity, health, and environmental sustainability.

**Abstract:**

A 39-day field experiment was conducted to assess the effect of grape marc (GM) feeding on sheep productivity, health, and environmental sustainability. Forty merino sheep were divided into two dietary groups, each having five replications (*n* = 4 sheep/replication). Experimental diet consisted of: (i) control: 55% lucerne hay + 40% wheat grain + 5% faba bean; (ii) GM treatment: control diet with 20% replaced by GM on a dry matter (DM) basis. The GM treatment contained 2–10% higher phytochemical contents than the control. The DMI from the GM treatment was 15% higher than the control (*p* < 0.001). No difference was found in sheep live weight gain, behaviour, and quality between groups (*p* > 0.05). No difference was found in total faecal production, faecal organic matter, and nitrogen contents (*p* > 0.05) and parasitic egg count. The GM treatment led to higher nitrogen intake (23.1 vs. 27.2 g/d) and faecal nitrogen excretion (6.3 vs. 8.7 g/d) compared to the control. Urinary creatinine, allantoin, and purine derivatives were lower in the GM treatment than control (*p* < 0.05). However, both groups had similar purine derivatives/DMI (i.e., indicator of rumen microbial protein synthesis efficiency; *p* > 0.05). Overall, the results showed that GM can replace 20% of the control ration to maintain sheep productivity, health, and environmental sustainability.

## 1. Introduction

Mitigating environmental pollution and improving both sheep productivity and welfare are the three main goals of sustainable sheep production. However, it is a challenge for sheep farmers in arid or semi-arid areas to meet these goals due to dry conditions and limited supply of home-grown forage, resulting in high prices of feed resources and, consequently, reduced profitability. To optimise sheep production, improve welfare and meet environmental goals, it is, therefore, important for sheep farmers to explore alternative and under-utilized feed resources, which are produced in large quantities and are non-edible for humans. Including waste products from fruit and vegetable industries in sheep diets may help to achieve these goals, by reducing the cost of feed rations [1] and providing biologically active phytochemicals to alter sheep metabolism [2,3]. One of the industry waste products that has recently attracted attention in the Australian livestock industries is grape marc (GM), which is also known as grape pomace, a waste by-product from the wine making industry that contains solid remains of grapes (seed and skin) after pressing for juice [4]. Grape marc has been used successfully as a supplement in forage-based dairy and beef production systems [1,5,6], sheep [6,7] and beef in feedlot production systems [8]. It has the potential to be widely used in forage-based sheep production systems in Australia. This is mainly because, as a waste product, GM is an inexpensive potential sheep feed source compared with traditional forage and southern Australia is a major grape growing region that has abundant supply of GM. Further, GM is produced in summer and autumn in Australia, when there is often a pasture shortage compared to sheep demand on the pasture-based production system in Australia [9]. Therefore, GM can be used as a potential feed source to fill the demand gap of pasture-based sheep production.

Earlier work showed GM contains condensed tannins [10] and polyphenol compounds with high antioxidant capacity [11]. Condensed tannin in GM may alter ruminal degradation and absorption of protein and lead to improved nitrogen (N) utilization efficiency and decreased urinary N in ruminant animals [12,13]. Urinary N, once excreted, will be hydrolysed immediately in soil into ammonia and nitrified into nitrate, which can subsequently pollute environment [14]. Therefore, decreased urinary N is beneficial to the environment. Further, Selby-Pham, et al. [15] and Chedea, et al. [16] showed feeding GM and grape skin extract improved pig and cow plasma polyphenol content and antioxidant capacity, respectively, compared with the control treatment. This indicates a potential health benefit in livestock through reducing the oxidative damage. However, the chemical components and concentrations contained in different GM vary in different grape varieties, compositions, and processing methods [6]. To the best of our knowledge, many of the previous studies that used GM were carried out in a controlled indoor feeding system for cattle [1] and sheep [17], where animals were fed individually. The majority of the studies used ensiled or dried GM, which required prior processing before feeding [1,7,8]. There have been limited feeding studies conducted to demonstrate the effects of inclusion of GM in a sheep production system/study on sheep performance, considering the overall physiological, environmental pollution and metabolic changes. Furthermore, understanding the interaction between diet components and GM is essential in sheep production systems to promote the use of waste products from the wine industry. The objective of the present study was to evaluate the effects of inclusion of GM in sheep diet on: (i) sheep productivity, through measuring dry matter (DM) intake, DM utilization, liveweight (LW) and body condition score (BCS) gain; (ii) animal welfare, through monitoring of behaviour and blood metabolites, and (iii) environmental sustainability, through estimation of urinary and faecal N in a typical forage-based feeding system in late summer/early autumn.

## 2. Materials and Methods

### 2.1. Experimental Site and Feeds

The study was conducted at The University of Melbourne, Dookie campus, northern Victoria, Australia (36°20′0″ S 145°42′0″ E). All procedures were conducted with approval from The University of Melbourne Animal Ethics Committee (application number 1814675.3). The study used a diet consisting of lucerne (*Medicago sativa*) hay, wheat (*Triticum aestivum*) grain and faba bean (*Vicia faba*) to feed sheep. These feeds were chosen because they are commonly used in forage-based sheep production systems in southern Australia. Fresh GM of *Vitis vinifera* L.cv. Shiraz was collected from The University of Melbourne, Dookie campus winery immediately after pressing on 11 March 2019 and stored at −20 °C prior to the commencement of the study on 27 March 2019. *Vitis vinifera* L.cv. Shiraz grapes harvested from a commercial vineyard were destemmed and fermented on skin for six days prior to pressing.

### 2.2. Animals, Diets, and Experimental Design 

The study used a total of 40 growing merino sheep (20 females and 20 males; 8 months of age), which were selected from the Dookie campus sheep flock with similar LW (31.6 ± 1.96 kg) and BCS (2.3 ± 0.37). The sheep were dewormed by giving 6 mL Cydectin plus tape (contains moxidectin) one week prior to the commencement of the study. Sheep were equally blocked into 2 dietary treatments of 20 sheep each (10 females and 10 males per dietary treatment), and then each group was further divided into 5 replication groups with 4 sheep per replication (i.e., 2 females and 2 males). Ten individual group pens were set up, each had 108 m^2^ (6 m × 18 m) to host 4 sheep. 

Water was offered ad libitum to each individual group. Feed allowance was offered at 10% above required maintenance energy level, calculated according to [18]. The two dietary treatments were: control (i.e., represents industry standard ration): 55% lucerne hay (*Medicago sativa*) + 40% wheat grain (*Triticum aestivum*) + 5% faba bean (*Vicia faba*) (DM basis); treatment: control diet with 20% ration being replaced by GM on a DM basis (GM diet). Frozen GM was left under room temperature overnight to thaw before daily feeding. The study was performed for 39 days, with a 14-day feed adaptation period and a 25-day treatment measurement period after (i.e., experimental day 1 to 25).

### 2.3. Feed Intake, Liveweight, and Body Condition Score Measurements

Feed was offered to sheep daily and DM intake was calculated by the difference between feed offered and refusals on a DM basis. Water intake was also measured daily, taking into consideration evaporation loss (a separate water trough with netting on top was used to measure evaporation loss). Feed samples were collected daily from each replication group, bulked weekly, and analysed for nutritive value via a pepsin-cellulase in vitro digestion method using the Australian Fodder Industry Association (AFIA [19] method 1.7R. Sheep LW and BCS were recorded after a 15 h fast on the first day of the adaptation period and experimental days 15, 26, and 39 (i.e., to quantify the carryover effect). The BCS was measured by two trained technicians on a 5-point scale with an increment of 0.5 according to the technical manual (EBELX, 2013).

### 2.4. Urine and Blood Sampling and Preparation

Mid-stream spot urine samples were randomly collected using the respiratory occlusion method from two sheep per replication group on experimental days 17 and 25. Samples were acidified to pH < 3 with concentrated sulphuric acid (98%, 18.4 M) to prevent ammonia volatilization.

### 2.5. Urine and Blood Sample Analysis

Urinary creatinine was analysed using a Cobas Integra 400 plus (Basel, Roche, Switzerland), and urinary uric acid and allantoins were determined using the uricase method and colorimetric method, respectively [20]. Purine derivatives excretion was estimated using the equation by [21].

$$\mathrm{Predicted}\;\mathrm{creatinine}\;\mathrm{coefficient}\;\left(\frac{\mathrm{mg}}{d.\;\mathrm{kg}}\right)==34.2\times W^{-0.104}$$

$$\mathrm{Predicted}\;\mathrm{purine}\;\mathrm{derivative}\left(s\right)\mathrm{excretion}\;\left(\frac{\mathrm{mmol}}{d}\right)\;=\frac{\left(\mathrm{predicted}\;\mathrm{creatinine}\;\mathrm{coefficient}\;\times \;W\right)}{113.12}\times \frac{\mathrm{urine}\;\mathrm{purine}\;\mathrm{derivatives}}{\mathrm{urine}\;\mathrm{creatinine}}$$

Blood samples were collected from two randomly selected sheep per replication group on measurement days 17 and 25. One 10 ml blood sample per sheep was collected from the jugular vein into a lithium heparin vacuette tube (BD 367526) using a 18G vacutainer needle (VACU NS2) and syringe (SYRI HS10). The blood was then centrifuged at 3500 rpm at 4 °C for 10 min to harvest plasma. Plasma urea N (PUN) and plasma glucose were analysed using a Cobas Integra 400 plus (Roche, Switzerland). Urinary N was predicted via the equation: Urinary N, g/d = 1.2 (L/d/kg LW) × PUN (g/L) × LW (kg) [22]. 

### 2.6. Total Faecal Output Analysis

Total faecal output was collected from 1 male sheep per replication group by attaching a faecal bag to the sheep. Sheep were crutched prior to bag installation and the faecal bags were used on experimental days 14–17 and 22–25. Faeces were sub sampled to conduct faecal egg counts, which were sent to the, Parasitology testing department, Department of Primary Industry, Menangle New South Wales 2568, Australia. Faeces were analysed for dry matter content using [19], (method 1.3R) and N content according to the Kjeldahl method [19] (method 1.4R). 

### 2.7. Animal Behaviour

Sheep behaviour was recorded through manually monitoring the sheep from a 5 m distance for eating, rumination, and idling activities 5 times a day on experimental day 10 within a time frame of 10:00–10:30, 12:40–13:40, 14:40–15:40, 16:30–17:00, and 18:00–18:30. Each group was monitored for 5 min with individual sheep behaviour recorded. 

### 2.8. Nutritive Value and Chemical Composition of Feeds

Subsamples of lucerne hay, wheat grain, faba bean, and GM with known fresh weight were oven-dried to constant weight at 65 °C for 48 h and then reweighed to determine percentage dry matter (% DM) using method 1.3R [20]. Ash content was determined using AFIA [19] method 1.10R. To calculate the organic matter (OM), ash % was subtracted from 100. Crude protein (CP) concentration was determined according to the Kjeldahl method (method 954.01) [23], and digestibility was determined using an in vitro pepsin-cellulose method, AFIA [19] method 1.7R. 

All phytochemical and antioxidant assays including total phenolics content (TPC), total flavonoid content (TFC), total tannin content (TTC), 1,1-diphenyl-2-picryl-hydrazyl (DPPH), 2,2’-azinobis-(3-ethylbenzothiazoline-6-sulfonic acid) (ABTS), and ferric reducing antioxidant power (FRAP) were run in triplicate and analyzed using a Multiskan® Go microplate photometer (Thermo Fisher Scientific, Waltham, MA, United States of America). The standard curves were plotted with R^2^ > 0.995.

### 2.9. Statistical Analysis

Data collected in this study were firstly tidied up in a Microsoft Office Excel spreadsheet and averaged per replication group for each parameter and then analysed via a one-way ANOVA using GenStat (version 16.1). The experimental unit was replication group, and the fixed factor was dietary treatment. In the case of faecal parameters statistical analysis, one missing value from the grape marc treatment group was taken into consideration, due to the loss of raw data during the experimental period.

## 3. Results

### 3.1. Nutritive Value and Chemical Composition of Feed

The nutritive value and chemical composition of the experimental feed are shown in Table 1. The DM content was lower for fresh GM (34.2%) compared with other feedstuffs. The mean CP concentration of GM (15.1% on DM basis) was higher than for wheat grain (13.4% on DM basis), but lower than the faba bean (25.6% on DM basis). Dry matter digestibility (DMD) of GM was considerably lower than the other feedstuffs (Table 1). 

GM had a greater concentration of antioxidants than other feedstuffs (Table 1). Replacing 20% of the control with GM (treatment) increased the concentration of bioactive compounds, i.e., 2 times higher total flavonoid, 7 times higher total phenolic, and 10 times higher total tannins concentration compared with control. 

### 3.2. Intake, Body Condition Score, and Liveweight 

The DMI was 15% higher in treatment than control (*p* < 0.001; Table 2). Sheep fed on GM diet drank approximately 10% less water than control sheep (*p* < 0.05). Feed conversion efficiency (LW gain/DMI), LW, and BCS gain were not different across diets (*p* > 0.05; Table 2). 

### 3.3. Faecal Output, Composition, and Egg Count

No effect was observed in the faecal output, organic matter, nitrogen content, and egg count between treatment and control groups (Table 3).

### 3.4. Nitrogen Balance and Purine Derivatives

Nitrogen intake was higher in the treatment group than the control (Table 4). However, the faecal nitrogen output and the ratio of faecal: urinary nitrogen was also higher in treatment than control (Table 4). In the case of urinary creatinine, allantoin, and purine derivatives treatment showed a lower value in comparison to the control (Table 4). The treatment group had no effect on PUN and glucose and purine derivatives excretion. However, purine derivatives excretion per DMI was only tentatively lower in treatment in comparison to control (Table 5).

### 3.5. Animal Behaviour

Sheep fed on the GM diet spent tentatively more time in eating than sheep fed on the control diet (Table 5). Feeding GM had no effect on ruminating and idling time (Table 5).

## 4. Discussion

### 4.1. Nutritive and Chemical Composition of Feedstuffs and Diets

Maximum water content from GM was extracted using a juicer prior to feeding. The GM used in the present study had a mean DM concentration of 34.2 ± 1.05%, OM concentration of 90.2 ± 0.55% of DM, and CP concentration of 15.1 ± 1.68% of DM. Compared to other studies, the mean OM concentration of GM used in the present study was at the lower end of the range for OM (93–94% of DM) and the mean CP at the higher end of the range for CP (4–15% of DM) reported in a couple of studies [2,24]. 

The mean DMD% of GM was 36.3 ± 1.65% of DM, approximately 60% lower than the mean DMD for wheat grain and faba bean and 20% lower than lucerne hay. The higher concentration of phytochemicals content (TPC, TFC, TTC, and DPPH) in the GM diet compared to the control diet restricted the dietary inclusion of GM up to 20%. However, the mean TTC concentration of the GM diet in the present study (25 g QE/ kg DM) is lower than the mean TTC (59 g QE/kg DM) of dried GP (at 20% inclusion) used in the study by Chikwanha, Moelich, Gouws, Muchenje, Nolte, Dugan and Mapiye [7].

### 4.2. Dry Matter Intake, Body Condition Score, and Liveweight

The higher DMI (17%) observed in the treatment group than the control (1.3 vs. 1.1 kg/sheep/d) may be attributed to enhanced palatability due to GM addition in the diet [7]. Consistent with the present study, Economides and Georghiades [25] found sheep on a 30% GM-an added diet had approximately 25% higher DMI than those on the control diet. Unlike the above study, the final LW and BCS were not significant between treatment and control in the present study due to large variation in SED. However, this non-significance may be due to offering 20% GM in the diet, whereas most studies noted that inclusion of dried grape pomace (DGP) beyond 10% is usually associated with an increase in DM intake and a decrease in feed efficiency [17,26,27]. As a result, a low proportion of GM in the diet could improve sheep productivity, while a high proportion of GM was more likely to have no or a negative effect in productivity due to the low digestibility and low energy value of GM. 

### 4.3. Faecal Output, Composition, and Egg Count

Sheep feeding on a GM diet consumed a significantly higher amount of feed, which explained the 30% higher dry faecal output in the treatment group compared with the control, with higher dry matter content, higher organic matter content, and higher N content. A range of 125–300 g DM faecal excretion was found [28]. The increased faecal output might be explained by the higher insoluble fibre content in the GM diet, and the presence of tannins further hinders the feedstuff digestibility. 

A faecal egg count is used for worm burdens monitoring in sheep. Merino sheep involved in this trial had been drenched with 6 mL Cydectin plus tape on 15th March 2019. No significant worm reproduction was observed for both diets, indicating both diets could retain the anthelmintic effect well. Niezen, et al. [29] reported that when sheep graze on tannins-rich legume crops, tannins could beneficially reduce the nematode infestation level and lower the number of parasite eggs in faeces, therefore providing a possible beneficial natural defence for ruminants. Similarly, Max, et al. [30] found that a quebracho tannin drench could significantly reduce the sheep total faecal egg count and thus could be considered as a natural anthelmintic with a potential to partially replace synthetic chemicals. 

### 4.4. Nitrogen Balance and Purine Derivatives

Sheep have a minimum protein requirement of 9–15%, depending on sheep age, growth conditions, and pregnancy status [31]. Sufficient protein supply can support maximum protein deposition and the highest possible efficiency. However, surplus protein would not benefit the performance but result in reduced N utilization efficiency and greater N excretion and changes carcass composition [32]. The GM greatly impacted on N balance. Faecal N for the GM diet increased by approximately 35% with a decrease of urinary N by 10%. A similar pattern was reported by Clifford [33], whereby sheep faecal N increased from 10.7 g/d to 14.5 g/d when 30% of the diet was replaced by GM, while urinary N decreased from 11.1 g/d to 9.0 g/d. A shift of N excretion from urine to faeces was observed, which may reflect a rumen function change [34]. The partitioning of surplus N away from urine to faeces may contribute to reduced nitrate leaching to ground water and nitrous oxide and ammonia emission to the atmosphere [35].

The overall N excretion decreased for the GM diet, indicating the retained N content increased. Inconsistent with the present results, Abarghuei, Rouzbehan and Alipour [24] observed that sheep feeding on a GM supplement diet had the same level of N intake, but it resulted in 170% higher faecal N and 18% lower urinary N. Clifford [33] also found a similar result that a 15% GM supplement in diet increased retained N by around 10%, while at 30% level GM supplementation the retained N dropped lower than that of control animals. CTs contained in GM could bind to protein and produce a tannins-protein insoluble complex that can hardly be digested and absorbed [36]. The lowered retained N in the 30% GM diet in Clifford’s study (2015) might be explained by the over-protection of tannins on protein, which made it difficult to be degraded in rumen. The relationship between retained and N intake represents protein quality [37].

The effect of GM diet on predicted purine derivatives (PD) excretion was not significant. Urinary PD excretion is known to have a strong correlation with microbial N availability and represents dietary N efficiency [21]. The increased urinary PD indicates the increased ruminal N availability in rumen and might be explained by the higher N intake for the GM diet. The relationship between predicted PD excretion and dry matter intake represents microbial energetic efficiency [38]. A tentative significant difference was found between the two group, while the GM diet showed a 15% decreased predicted PD:DMI ratio, indicating relatively lower ruminal microbial protein efficiency. Consistent with the present study, Abarghuei, Rouzbehan and Alipour [24] found a lower ruminal microbial protein synthesis in the GM diet, and Yáñez-Ruiz and Molina-Alcaide [39] also reported a decreased microbial protein level in sheep fed olive cake (16.7 g CT/kg DM). The decreased microbial protein synthesis efficiency could be explained by the effect of tannins on ruminal protein metabolism as discussed above. Tannins could bind to plant protein, reduce the microbial enzymes activity, and reduce the proteolytic bacteria growth rate, thus ultimately leading to decreased microbial protein efficiency. 

### 4.5. Animal Behaviour

Study in animal behaviour is of great importance for model development to support livestock husbandry management that would show when and how adjustment of feeding would be needed for improvement in animal productivity [40]. Both dietary fibre concentration and composition could influence behavioural activities, especially rumination. In this study, merino sheep consuming a GM diet exhibited longer chewing times, which can be explained by the high level of neutral detergent fibre in fresh GM. Additionally, the higher apparent feed intake for the GM supplement diet might also be a part of the reason for longer chewing time. 

The present study has shown that sheep for both diets spent the most time idling, eating was the next, and the least time was spent ruminating. Dellow and Barry [41] reported a contradictory behaviour pattern whereby sheep (3.5-year-old) being fed chaffed lucerne hay spent 8.3 h ruminating and 3.7 h eating in a 24 h period. The feeding method might be responsible for the altered pattern, as Dellow and Barry [41] used hourly feeding, while once-a-day feeding was applied in the present study. According to Dellow and Barry [41], 3.7 h ruminating and 2.0 h eating took place in the day period (06.00–18.00), and 4.6 h ruminating and 1.7 h eating occurred in the night period (18.00–06.00). The total amount of chewing time (eating plus ruminating) was 7 h and 6.3 h during the day and night, respectively. That indicated sheep rumination frequency increased in the night period, while behaviour monitoring was only conducted in the day period in the present study; using data collected from 3.5 h in the day period to estimate the whole day behaviour may not be accurate enough. Further study with an increased measuring time and period should be considered.

## 5. Conclusions

The GM addition in the sheep diet had a compensating effect on sheep production. Furthermore, efficient utilization of nitrogen not only provides possible nutrients to maintain body weight but also reduces environment pollution from urinary nitrogen loss. Overall, inclusion of grape marc might be a better idea in the ruminant diet, especially in the wine producing areas in the world.

## Figures and Tables

**Table 1:** Nutritive value and chemical composition of wheat grain, faba bean, lucerne hay, grape marc, and experimental diets.

	Control			Total Diet	Treatment				Total Diet
Items	Wheat Grain	Faba Bean	Lucerne Hay		Wheat Grain	Faba Bean	Lucerne Hay	Grape Marc	
DM%	95.6 ± 0.71	96.8 ± 0.13	97.5 ± 0.69	96.7	95.4 ± 1.44	96.3 ± 1.14	97.1 ± 0.60	34.2 ± 1.05	84.0
DOM%	98.0 ± 0.20	96.4 ± 0.38	92.3 ± 0.41	94.8	98.0 ± 0.11	96.6 ± 0.09	92.6 ± 0.50	90.2 ± 0.55	94.0
Ash%	2.0 ± 0.20	3.6 ± 0.38	7.7 ± 0.41	5.2	2.0 ± 0.11	3.5 ± 0.09	7.4 ± 0.50	9.8 ± 0.55	6.0
CP%	13.6 ± 0.51	24.7 ± 0.44	11.6 ± 0.84	13.0	13.4 ± 0.44	25.6 ± 0.84	11.2 ± 1.66	15.1 ± 1.68	13.2
Pepsin-Cellulase Digestibility									
DMD%	94.8 ± 0.20	89.4 ± 1.20	44.8 ± 3.31	67.0	94.9 ± 0.23	89.2 ± 0.99	48.2 ± 3.43	36.3 ± 1.65	62.4
OMD%	91.9 ± 4.25	86.1 ± 4.23	38.9 ± 6.31	62.4	93.9 ± 0.88	86.9 ± 2.30	41.0 ± 5.33	29.5 ± 1.53	57.5
DOMD%	92.1 ± 0.98	83.0 ± 3.89	35.9 ± 5.75	60.7	92.1 ± 0.79	83.9 ± 2.18	38.0 ± 5.03	26.6 ± 2.18	51.7
Antioxidant Compounds									
TPC1	0.4 ± 0.01	1.9 ± 0.11	0.5 ± 0.01	0.5	0.6 ± 0.03	2.3 ± 0.11	0.5 ± 0.02	16.0 ± 0.46	3.7
TFC2	2.0 ± 0.09	6.1 ± 0.12	1.9 ± 0.08	2.1	1.6 ± 0.17	14.9 ± 0.48	1.7 ± 0.02	14.9 ± 0.45	4.9
TTC3	3.2 ± 3.1	6.0 ± 5.9	1.1 ± 1.1	2.2	8.2 ± 1.03	5.0 ± 0.12	1.0 ± 0.08	22.1 ± 4.7	24.8
DPPH4	0.1 ± 0.00	2.3 ± 0.04	0.2 ± 0.00	0.2	0.1 ± 0.00	2.2 ± 0.19	0.1 ± 0.00	21.7 ± 0.81	4.5
FRAP4	0.1 ± 0.05	0.3 ± 0.24	0.0 ± 0.01	0.1	0.0 ± 0.01	0.3 ± 0.08	0.0 ± 0.01	1.0 ± 0.48	0.2

All assays based on dry matter. The control diet consists of 55% lucerne hay, 40% wheat grain, and 5% faba bean; treatment diet has 20% control diet being replaced by grape marc. DM—dry matter; DOM—digestible organic matter; CP—crude protein; DMD—dry matter digestibility; OMD—organic matter digestibility; DOMD—digestible organic matter in dry matter. 1 Total phenolic content is expressed as g of gallic acid equivalent (GAE)/kg DM. 2 Total flavonoid content is expressed as g of quercetin equivalents (QE)/kg DM. 3 Total tannins content is expressed as g of catechin equivalents (CE)/kg DM. 4 Antioxidant activities are expressed as g of ascorbic acid equivalent (AAE)/kg DM.

**Table 2:** Effects of feeding a diet containing 20% grape marc on productivity of merino sheep.

Parameters	Control	Treatment	SED	*p*-Value
Fresh matter intake, kg/head/d	1.14	1.79	0.24	0.025
Dry matter intake, kg/head/d	1.11	1.30	0.02	< 0.001
Water intake, kg/head/d	3.00	2.72	0.11	0.030
Liveweight gain, g/head/d	33.0	5.0	22.5	0.248
BCS change, unit/head p/d	0.02	0.02	0.01	0.719
Feed conversion efficiency, g/kg	30.0	3.5	18.0	0.181

The control diet consists of 55% lucerne hay, 40% wheat grain, and 5% faba bean; treatment diet has 20% control diet being replaced by grape marc; SED: standard error of deviation.

**Table 3:** Effects of feeding a diet containing 20% grape marc on faecal output, faecal composition, and egg count of merino sheep.

Parameters	Control	Treatment	SED	*p*-Value
Fresh faecal output, kg/sheep/d	0.8	0.9	0.10	0.232
Dry faecal output, kg/sheep/d	0.3	0.4	0.04	0.075
Faecal dry matter, %	35.9	39.7	1.63	0.052
Faecal organic matter, %	91.4	92.0	0.37	0.132
Faecal nitrogen content, %	2.3	2.5	0.10	0.174
Faecal egg count				
Strongyle, epg	16	8	17.9	0.667
Nematodirus, epg	0	16	16.0	0.347
Tapeworm	-	-	-	-
Coccidia	+	+	-	-

Tapeworm or coccidial eggs are present; they are scored as: + = low numbers; - = absent; epg = eggs per gram of faeces; SED: standard error of deviation.

**Table 4:** Effects of feeding a diet containing 20% grape marc on predicted nitrogen balance, plasma metabolites, and urinary purine derivatives of merino sheep.

Parameters	Control	Treatment	SED	*p*-Value
Nitrogen intake, g/sheep/d	23.1	27.2	0.50	<0.001
Faecal nitrogen output, g/sheep/d	6.3	8.7	1.00	0.047
Urinary nitrogen output, g/sheep/d	8.2	7.3	0.59	0.138
Retained nitrogen, g/sheep/d	8.6	11.2	1.33	0.091
Retained/nitrogen intake, g/g	0.4	0.4	0.05	0.509
Faecal nitrogen/urinary nitrogen, g/g	0.8	1.2	0.14	0.024
Plasma urea nitrogen, g/L	0.2	0.2	0.01	0.146
Plasma glucose, mmol/L	4.1	4.5	0.43	0.424
Urinary creatinine, mmol/L	10.3	7.1	1.22	0.033
Urinary allantoin, mmol/L	14.0	10.0	1.72	0.046
Uric acid, mmol/L	1.6	1.0	0.32	0.088
Purine derivatives, mmol/L	15.6	10.9	1.82	0.034
Purine derivatives excretion, mmol/sheep/d	11.0	11.0	0.86	0.939
Purine derivatives excretion: dry matter intake, mmol/g	9.96	8.43	0.73	0.069

Retained nitrogen (g/d) = nitrogen intake (g/d) – (faecal nitrogen(g/d) + urinary nitrogen (g/d)). Purine derivatives (mmol/l) = allantoin (mmol/l) + uric acid (mmol/l); SED: standard error of deviation.

**Table 5:** Effects of feeding a diet containing 20% grape marc on behaviour of merino sheep.

Parameter	Control	Treatment	SED	*p*-Value
Eating, min/3.5 hr/group	59.5	79.5	9.15	0.060
Rumination, min/3.5 hr/group	17.0	17.0	6.09	1.000
Idling, min/3.5 hr/group	133.5	113.5	12.70	0.154

SED: standard error of deviation.

## Data Availability

Not applicable.
